# 3-(4-Ethoxy­benzo­yl)propionic acid

**DOI:** 10.1107/S1600536808034429

**Published:** 2008-10-25

**Authors:** Sajid Ali, Aurangzeb Hasan, Nasim Hasan Rama, Amir Badshah, Ales Ruczika

**Affiliations:** a Department of Chemistry, Quaid-i-Azam University, Islamabad 45320, Pakistan; b Department of General and Inorganic Chemistry, Faculty of Chemical Technology, University of Pardubice, Nam. Cs. Legii’ 565, 53210 Pardubice, Czech Republic

## Abstract

The title compound, C_12_H_14_O_4_, is an important inter­mediate in the synthesis of biologically active heterocyclic compounds. In the crystal structure, inter­molecular O—H⋯O and C—H⋯O hydrogen bonds link the mol­ecules. There are also C—H⋯π contacts between the benzene ring and the methyl­ene groups.

## Related literature

For general background, see: Hashem *et al.* (2007 ▶); Husain *et al.* (2005 ▶). For bond-length data, see: Allen *et al.* (1987 ▶).
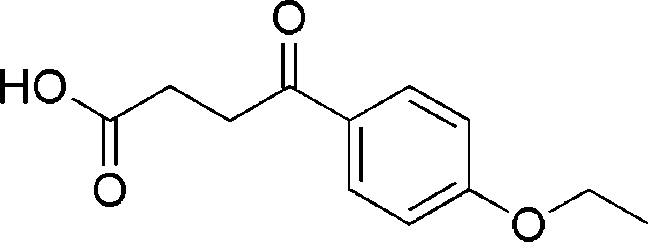


## Experimental

### 

#### Crystal data

C_12_H_14_O_4_
*M* *_r_* = 222.23
Triclinic, 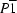

*a* = 7.8371 (3) Å
*b* = 8.7399 (5) Å
*c* = 9.8140 (5) Å
α = 106.993 (4)°
β = 107.541 (4)°
γ = 107.142 (4)°
*V* = 556.34 (6) Å^3^
*Z* = 2
Mo *K*α radiation
μ = 0.10 mm^−1^
*T* = 150 (1) K
0.34 × 0.32 × 0.31 mm

#### Data collection

Bruker–Nonius KappaCCD area-detector diffractometer
Absorption correction: Gaussian (Coppens, 1970 ▶) *T*_min_ = 0.964, *T*_max_ = 0.987
8993 measured reflections
2508 independent reflections
2089 reflections with *I* > 2σ(*I*)
*R*_int_ = 0.059

#### Refinement

*R*[*F* ^2^ > 2σ(*F* ^2^)] = 0.047
*wR*(*F* ^2^) = 0.123
*S* = 1.11
2508 reflections
145 parameters
H-atom parameters constrained
Δρ_max_ = 0.22 e Å^−3^
Δρ_min_ = −0.24 e Å^−3^

### 

Data collection: *COLLECT* (Hooft, 1998 ▶); cell refinement: *COLLECT* and *DENZO* (Otwinowski & Minor, 1997 ▶); data reduction: *COLLECT* and *DENZO*; program(s) used to solve structure: *SIR92* (Altomare *et al.*, 1994 ▶); program(s) used to refine structure: *SHELXL97* (Sheldrick, 2008 ▶); molecular graphics: *PLATON* (Spek, 2003 ▶); software used to prepare material for publication: *SHELXL97*.

## Supplementary Material

Crystal structure: contains datablocks I, global. DOI: 10.1107/S1600536808034429/hk2555sup1.cif

Structure factors: contains datablocks I. DOI: 10.1107/S1600536808034429/hk2555Isup2.hkl

Additional supplementary materials:  crystallographic information; 3D view; checkCIF report

## Figures and Tables

**Table 1:** Hydrogen-bond geometry (Å, °)

*D*—H⋯*A*	*D*—H	H⋯*A*	*D*⋯*A*	*D*—H⋯*A*
O2—H2⋯O1^i^	0.82	1.85	2.664 (3)	172
C2—H2*B*⋯O3^ii^	0.97	2.44	3.386 (3)	165
C11—H11*B*⋯O3^iii^	0.97	2.50	3.445 (3)	166
C3—H3*B*⋯*Cg*1^iv^	0.97	2.66	3.528 (3)	150
C11—H11*A*⋯*Cg*1^v^	0.97	2.84	3.679 (3)	145

Symmetry codes: (i) 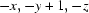; (ii) 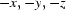; (iii) 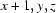; (iv) 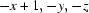; (v) 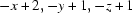. *Cg*1 is the centroid of the phenyl ring.

**Fig. 1.**
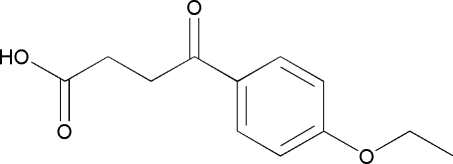
The molecular structure of the title molecule, with the atom-numbering scheme.

**Fig. 2.**
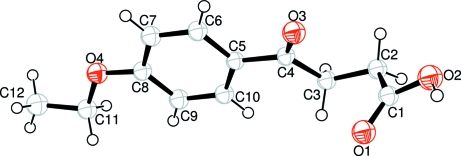
A partial packing diagram. Hydrogen bonds are shown as dashed lines.

**Fig. 3.**
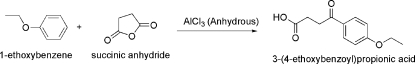
The formation of the title compound.

## supplementary crystallographic information

### Comment

Benzoyl propionic acids are important intermediates in heterocyclic chemistry and have been used for the synthesis of various biologically active five -membered heterocyles such as butenolides, pyrrolones (Husain *et al.*, 2005), oxadiazoles and triazoles (Hashem *et al.*,  2007). In view of the versatility of these compounds, we synthesized the title compound and reported herein its crystal structure.

In the title compound (Fig. 1), the bond lengths (Allen *et al.*, 1987) and angles are within normal ranges . O4, C2, C3, C4, C11 and C12 atoms are -0.011 (3), 0.162 (4), 0.189 (4), -0.09 (3), -0.143 (3) and -0.151 (3) Å away from the phenyl plane, respectively.

In the crystal structure, intermolecular O-H···O and C-H···O hydrogen bonds (Table 1) link the molecules (Fig. 2), in which they may be effective in the stabilization of the structure. There also exist C—H···π contacts (Table 1) between the phenyl ring and the methylene groups.

### Experimental

The title compound was synthesized by the condensation of succinic anhydride (2 g, 20 mmol) with 1-ethoxybenzene (10 ml) in the presence of alumium chloride (6 g, 42 mmol). The reaction mixture was refluxed for 4 h. After completion of the reaction, excess solvent (Anisol) was removed by steam distillation. The resultant solid product was purified by dissolving it in sodium hydroxide solution (5%, *w*/*v*), filtering followed by addition of hydrochloric acid. The obtained solid mass was filtered, washed with cold water, dried and crystallized from methanol (yield; 67%, m.p. 404-405 K).

### Crystal data

**Table:**

C_12_H_14_O_4_	*Z* = 2
*M**_r_* = 222.23	*F*(000) = 236
Triclinic, *P*1	*D*_x_ = 1.327 Mg m^−^^3^
Hall symbol: -P 1	Melting point: 404(1) K
*a* = 7.8371 (3) Å	Mo *K*α radiation, λ = 0.71073 Å
*b* = 8.7399 (5) Å	Cell parameters from 9044 reflections
*c* = 9.8140 (5) Å	θ = 1–27.5°
α = 106.993 (4)°	µ = 0.10 mm^−^^1^
β = 107.541 (4)°	*T* = 150 K
γ = 107.142 (4)°	Block, colorless
*V* = 556.34 (6) Å^3^	0.34 × 0.32 × 0.31 mm

### Data collection

**Table:**

Bruker–Nonius KappaCCD area-detector diffractometer	2508 independent reflections
Radiation source: fine-focus sealed tube	2089 reflections with *I* > 2σ(*I*)
graphite	*R*_int_ = 0.059
Detector resolution: 9.091 pixels mm^-1^	θ_max_ = 27.5°, θ_min_ = 2.4°
φ and ω scans	*h* = −10→10
Absorption correction: gaussian (Coppens, 1970)	*k* = −11→11
*T*_min_ = 0.964, *T*_max_ = 0.987	*l* = −12→12
8993 measured reflections	

### Refinement

**Table:**

Refinement on *F*^2^	Primary atom site location: structure-invariant direct methods
Least-squares matrix: full	Secondary atom site location: difference Fourier map
*R*[*F*^2^ > 2σ(*F*^2^)] = 0.047	Hydrogen site location: inferred from neighbouring sites
*wR*(*F*^2^) = 0.123	H-atom parameters constrained
*S* = 1.11	*w* = 1/[σ^2^(*F*_o_^2^) + (0.0401*P*)^2^ + 0.2871*P*] where *P* = (*F*_o_^2^ + 2*F*_c_^2^)/3
2508 reflections	(Δ/σ)_max_ < 0.001
145 parameters	Δρ_max_ = 0.22 e Å^−^^3^
0 restraints	Δρ_min_ = −0.24 e Å^−^^3^

### Special details

**Table:**

Geometry. All e.s.d.'s (except the e.s.d. in the dihedral angle between two l.s. planes) are estimated using the full covariance matrix. The cell e.s.d.'s are taken into account individually in the estimation of e.s.d.'s in distances, angles and torsion angles; correlations between e.s.d.'s in cell parameters are only used when they are defined by crystal symmetry. An approximate (isotropic) treatment of cell e.s.d.'s is used for estimating e.s.d.'s involving l.s. planes.
Refinement. Refinement of *F*^2^ against ALL reflections. The weighted *R*-factor *wR* and goodness of fit *S* are based on *F*^2^, conventional *R*-factors *R* are based on *F*, with *F* set to zero for negative *F*^2^. The threshold expression of *F*^2^ > σ(*F*^2^) is used only for calculating *R*-factors(gt) *etc*. and is not relevant to the choice of reflections for refinement. *R*-factors based on *F*^2^ are statistically about twice as large as those based on *F*, and *R*- factors based on ALL data will be even larger.

### Fractional atomic coordinates and isotropic or equivalent isotropic displacement parameters (Å^2^)

**Table:**

	*x*	*y*	*z*	*U*_iso_*/*U*_eq_	
O1	0.19232 (17)	0.45879 (16)	0.01166 (16)	0.0387 (3)	
O2	−0.11942 (17)	0.27131 (16)	−0.14840 (16)	0.0419 (3)	
H2	−0.1388	0.3584	−0.1118	0.050*	
O3	0.21830 (18)	0.16047 (18)	0.13387 (15)	0.0373 (3)	
O4	1.07552 (17)	0.25792 (17)	0.51668 (14)	0.0352 (3)	
C1	0.0717 (2)	0.3129 (2)	−0.08709 (19)	0.0287 (3)	
C2	0.1221 (2)	0.1655 (2)	−0.15467 (19)	0.0295 (3)	
H2A	0.1061	0.1493	−0.2606	0.035*	
H2B	0.0308	0.0577	−0.1615	0.035*	
C3	0.3310 (2)	0.1968 (2)	−0.05809 (19)	0.0279 (3)	
H3A	0.4210	0.3134	−0.0364	0.033*	
H3B	0.3658	0.1115	−0.1184	0.033*	
C4	0.3542 (2)	0.1827 (2)	0.09544 (18)	0.0261 (3)	
C5	0.5470 (2)	0.1981 (2)	0.19915 (18)	0.0258 (3)	
C6	0.5593 (2)	0.1507 (2)	0.32477 (19)	0.0302 (4)	
H6	0.4458	0.1043	0.3385	0.036*	
C7	0.7366 (3)	0.1717 (2)	0.4281 (2)	0.0322 (4)	
H7	0.7427	0.1394	0.5111	0.039*	
C8	0.9075 (2)	0.2414 (2)	0.40859 (19)	0.0293 (3)	
C9	0.8975 (2)	0.2867 (2)	0.2828 (2)	0.0326 (4)	
H9	1.0105	0.3314	0.2679	0.039*	
C10	0.7176 (2)	0.2637 (2)	0.1798 (2)	0.0306 (4)	
H10	0.7107	0.2933	0.0952	0.037*	
C11	1.2575 (2)	0.3431 (2)	0.5103 (2)	0.0338 (4)	
H11A	1.2781	0.4618	0.5209	0.041*	
H11B	1.2554	0.2783	0.4105	0.041*	
C12	1.4192 (3)	0.3482 (3)	0.6432 (2)	0.0424 (4)	
H12A	1.4192	0.4118	0.7412	0.051*	
H12B	1.5439	0.4059	0.6429	0.051*	
H12C	1.3981	0.2300	0.6308	0.051*	

### Atomic displacement parameters (Å^2^)

**Table:**

	*U*^11^	*U*^22^	*U*^33^	*U*^12^	*U*^13^	*U*^23^
O1	0.0274 (6)	0.0308 (6)	0.0479 (8)	0.0141 (5)	0.0076 (5)	0.0101 (6)
O2	0.0270 (6)	0.0365 (7)	0.0490 (8)	0.0156 (5)	0.0077 (6)	0.0076 (6)
O3	0.0318 (6)	0.0519 (8)	0.0351 (7)	0.0193 (6)	0.0182 (5)	0.0212 (6)
O4	0.0299 (6)	0.0454 (7)	0.0319 (6)	0.0157 (5)	0.0100 (5)	0.0213 (6)
C1	0.0257 (8)	0.0316 (8)	0.0305 (8)	0.0125 (7)	0.0099 (7)	0.0170 (7)
C2	0.0294 (8)	0.0327 (8)	0.0249 (8)	0.0138 (7)	0.0094 (6)	0.0116 (6)
C3	0.0294 (8)	0.0308 (8)	0.0278 (8)	0.0166 (7)	0.0129 (7)	0.0130 (7)
C4	0.0292 (8)	0.0233 (7)	0.0267 (8)	0.0121 (6)	0.0132 (6)	0.0091 (6)
C5	0.0297 (8)	0.0256 (7)	0.0248 (8)	0.0144 (6)	0.0118 (6)	0.0110 (6)
C6	0.0321 (8)	0.0338 (8)	0.0308 (8)	0.0157 (7)	0.0172 (7)	0.0155 (7)
C7	0.0369 (9)	0.0364 (9)	0.0290 (8)	0.0169 (7)	0.0154 (7)	0.0178 (7)
C8	0.0311 (8)	0.0295 (8)	0.0272 (8)	0.0151 (7)	0.0095 (7)	0.0122 (7)
C9	0.0293 (8)	0.0389 (9)	0.0347 (9)	0.0145 (7)	0.0156 (7)	0.0197 (8)
C10	0.0322 (8)	0.0382 (9)	0.0286 (8)	0.0170 (7)	0.0146 (7)	0.0192 (7)
C11	0.0305 (8)	0.0383 (9)	0.0297 (9)	0.0135 (7)	0.0103 (7)	0.0141 (7)
C12	0.0325 (9)	0.0505 (11)	0.0374 (10)	0.0138 (8)	0.0088 (8)	0.0196 (9)

### Geometric parameters (Å, °)

**Table:**

O2—H2	0.8200	C5—C10	1.384 (2)
O3—C4	1.2156 (19)	C6—C7	1.373 (2)
O4—C8	1.3552 (19)	C6—H6	0.9300
O4—C11	1.431 (2)	C7—H7	0.9300
C1—O1	1.214 (2)	C8—C7	1.394 (2)
C1—O2	1.3210 (19)	C8—C9	1.390 (2)
C1—C2	1.488 (2)	C9—H9	0.9300
C2—H2A	0.9700	C10—C9	1.381 (2)
C2—H2B	0.9701	C10—H10	0.9300
C3—C2	1.517 (2)	C11—C12	1.500 (2)
C3—C4	1.508 (2)	C11—H11A	0.9700
C3—H3A	0.9700	C11—H11B	0.9700
C3—H3B	0.9700	C12—H12A	0.9599
C5—C4	1.487 (2)	C12—H12B	0.9600
C5—C6	1.397 (2)	C12—H12C	0.9600
			
C1—O2—H2	109.5	C5—C6—H6	119.6
C8—O4—C11	117.96 (13)	C6—C7—C8	120.05 (15)
O1—C1—O2	122.54 (15)	C6—C7—H7	120.0
O1—C1—C2	124.21 (14)	C8—C7—H7	120.0
O2—C1—C2	113.22 (14)	O4—C8—C9	124.30 (15)
C1—C2—C3	112.93 (14)	O4—C8—C7	115.80 (14)
C1—C2—H2A	109.0	C9—C8—C7	119.89 (15)
C3—C2—H2A	109.0	C10—C9—C8	119.16 (15)
C1—C2—H2B	109.0	C10—C9—H9	120.4
C3—C2—H2B	108.9	C8—C9—H9	120.4
H2A—C2—H2B	107.8	C9—C10—C5	121.76 (15)
C4—C3—C2	111.95 (13)	C9—C10—H10	119.2
C4—C3—H3A	109.1	C5—C10—H10	119.1
C2—C3—H3A	109.1	O4—C11—C12	107.39 (14)
C4—C3—H3B	109.4	O4—C11—H11A	110.2
C2—C3—H3B	109.4	C12—C11—H11A	110.3
H3A—C3—H3B	107.9	O4—C11—H11B	110.3
O3—C4—C5	120.78 (14)	C12—C11—H11B	110.2
O3—C4—C3	120.57 (14)	H11A—C11—H11B	108.5
C5—C4—C3	118.65 (13)	C11—C12—H12A	109.4
C10—C5—C6	118.30 (15)	C11—C12—H12B	109.5
C10—C5—C4	122.60 (14)	H12A—C12—H12B	109.5
C6—C5—C4	119.06 (14)	C11—C12—H12C	109.5
C7—C6—C5	120.82 (15)	H12A—C12—H12C	109.5
C7—C6—H6	119.6	H12B—C12—H12C	109.5

### Hydrogen-bond geometry (Å, °)

**Table:**

*D*—H···*A*	*D*—H	H···*A*	*D*···*A*	*D*—H···*A*
O2—H2···O1^i^	0.82	1.85	2.664 (3)	172
C2—H2B···O3^ii^	0.97	2.44	3.386 (3)	165
C11—H11B···O3^iii^	0.97	2.50	3.445 (3)	166
C3—H3B···Cg1^iv^	0.97	2.66	3.528 (3)	150
C11—H11A···Cg1^v^	0.97	2.84	3.679 (3)	145

Symmetry codes: (i) −*x*, −*y*+1, −*z*; (ii) −*x*, −*y*, −*z*; (iii) *x*+1, *y*, *z*; (iv) −*x*+1, −*y*, −*z*; (v) −*x*+2, −*y*+1, −*z*+1.
